# Associations between 1930s HOLC grades and estimated population burden of cardiovascular disease risk factors in 2020

**DOI:** 10.1093/pnasnexus/pgae301

**Published:** 2024-08-09

**Authors:** Hanxue Wei, Benjamin R Spoer, Andrea R Titus, Taylor M Lampe, Marc N Gourevitch, Jacob W Faber, Steven J Korzeniewski, Samantha J Bauer, Lorna E Thorpe

**Affiliations:** Department of Population Health, New York University Grossman School of Medicine, New York, NY 10016, USA; Department of Population Health, New York University Grossman School of Medicine, New York, NY 10016, USA; Department of Population Health, New York University Grossman School of Medicine, New York, NY 10016, USA; Department of Population Health, New York University Grossman School of Medicine, New York, NY 10016, USA; Department of Population Health, New York University Grossman School of Medicine, New York, NY 10016, USA; Robert F. Wagner School, New York University, New York, NY 10003, USA; Department of Sociology, New York University, New York, NY 10003, USA; Department of Family Medicine and Public Health Sciences, Wayne State University School of Medicine, Detroit, MI 48201, USA; Department of Family Medicine and Public Health Sciences, Wayne State University School of Medicine, Detroit, MI 48201, USA; Department of Population Health, New York University Grossman School of Medicine, New York, NY 10016, USA

**Keywords:** redlining, HOLC, cardiovascular risk factor, IPW

## Abstract

Studies have recently begun to explore the potential long-term health impacts of homeownership policies implemented in the New Deal era. We investigated the association between assigned grades of lending risk by the Home Owners’ Load Corporation (HOLC) maps from the 1930s and present-day prevalence of three cardiovascular risk factors (diabetes and obesity in 2020, and hypertension in 2019), estimated at the census tract level in the United States. To minimize potential confounding, we adjusted for sociodemographic data from the time period when HOLC maps were made. We calculated propensity scores (predicted probability of receiving a HOLC grade) and created a pseudo-population using inverse probability weighting. We then employed marginal structural models to estimate prevalence differences comparing A vs. B, B vs. C, and C vs. D HOLC grades. Adjusting only for regions, a less desirable HOLC grade was associated with higher estimated prevalence rates of present-day cardiovascular risk factors; however, most differences were no longer significant after applying propensity score methods. The one exception was that the prevalence of diabetes, hypertension, and obesity were all higher in C vs. B graded census tracts, while no differences were observed for C and D and A and B comparisons. These results contribute to a small body of evidence that suggests historical “yellowlining” (as C grade was in color yellow) may have had persistent impacts on neighborhood-level cardiovascular risk factors 80 years later.

**Significance Statement**

Our findings highlight that mortgage lending risk assessment practices in the 1930s may have had differential persistent impacts on cardiovascular health up to eight decades later. Findings suggest that policy interventions like neighborhood reinvestment may need to address the potential impact not only of historical “redlining” practices but of “yellowlining” as well. This research applies methods to account for potential differences in population composition prior to the assignment of lending grades, allowing us to better examine the potential neighborhood-level causal impacts of historical discriminatory housing policies on cardiovascular health disparities.

## Introduction

As part of the New Deal, the federal government founded the Home Owners’ Loan Corporation (HOLC) and the Federal Housing Administration (FHA) in 1933 and 1934, respectively. HOLC was designed to reduce high foreclosure rates in the United States during the Great Depression, and FHA aimed to expand homeownership and spur employment. From 1935 to 1940, as part of its City Survey Program, HOLC created Residential Security Maps (also known as “HOLC maps”).

These maps categorized neighborhoods into four color-coded grades based on HOLC's assessment of the mortgage lending risks. This grading system reflected characteristics of the housing stock such as building age, but also factors such as the race, ethnicity, and immigration status of neighborhood residents (1). Areas colored in green were graded “A” or “best,” areas in blue were graded “B” or “still desirable,” yellow for grade “C” or “definitely declining,” and red for grade “D” or “hazardous.” Historical documents are clear that the greater the proportion of Black (then referred to as “Negro”) residents a neighborhood had, the less desirable its HOLC grade, and indeed, D-graded areas had the highest concentrations of Black residents (2). Most of the D-graded areas today remain low-income and racial/ethnic minority neighborhoods (3). These areas also tend to have more vacant houses, which is a common sign of neighborhood abandonment (4, 5). As HOLC mapmakers used information collected from local real estate and lending professionals to create these maps (4), the maps reflected existing discriminatory lending practices and racial biases in the housing market. Scholars have argued that the HOLC maps both reflected and contributed further to discriminatory lending practices at the time (2, 6, 7), and those discriminatory practices in the housing market contributed to continued disinvestment in low-income and racial/ethnic minority neighborhoods, especially for Black residents. The practice of restricting Black home ownership and investment in predominantly Black neighborhoods existed before HOLC, but became known as “redlining” due to the red areas of HOLC's maps, reflecting that potential borrowers were denied access to credit due to the demographic composition of their neighborhood (1, 8). In 1980, Jackson first highlighted the HOLC maps as examples of residential redlining (8, 9). In 2016, the Mapping Inequality project digitized HOLC's maps (10), and since then investigators have increasingly examined the persistent health, social, and economic impacts of redlining by HOLC and other federal agencies on local communities (11).

HOLC maps have been linked to present-day inequities in housing and credit access for minorities (12). Researchers have also found evidence indicating a causal relationship between redlining and reduced socioeconomic mobility (1). Disinvestment stemming from redlining has been hypothesized as one mechanism behind observed associations with persistent lack of access to quality education (13), food access (14), as well as increased racial segregation (1, 15). It has also been associated with adverse environmental health risks such as increased exposure to urban heat (16). Links between redlining and adverse health outcomes have also been widely investigated, including but not limited to chronic diseases, maternal and infant health, mental health, and heat-related illness (11). Redlining is hypothesized to negatively affect the health of minoritized communities through several key pathways: increased exposure to environmental and social hazards, such as urban heat islands (16) and inadequate education resources (13); and reduced access to mitigating resources, such as health services, that could alleviate these hazards (17). Additionally, areas graded as C and D predominantly housed residents of color, particularly Black residents, reinforcing racialized economic segregation and its associated health disparities (18).

Cardiovascular diseases contribute significantly to reduced quality and duration of life and are a major cause of death globally (19). Studying the risk factors for cardiovascular disease provides critical insights into the interrelated influence of lifestyle, genetics, environment, and policy factors on health. Emerging evidence links redlining and present-day health disparities, and recent research has found associations between historical HOLC maps and present-day cardiovascular disease risk factors at the neighborhood-level (20, 21) and individual-level (6, 22). For instance, Mujahid et al. (6) found that such associations were more pronounced among Black residents than White residents within the same neighborhoods. It thus appears plausible that lending practices influenced by the grades on HOLC maps nearly a century ago have led to present-day, intergenerational disparities in the prevalence of cardiovascular disease risk factors, especially given recent evidence of multigenerational “neighborhood effects” (23).

However, the application of causal inference techniques remains limited in this research area, with a notable lack of adjustment for historical sociodemographic factors that may confound the association between HOLC grades and contemporary prevalence of health outcomes. Prior neighborhood-level studies predominantly adjusted for recent sociodemographic variables, which likely lie on the pathway between 1940s sociodemographics and present-day health outcomes (24, 25). Nardone et al. (25, 26) utilized propensity score methods to investigate redlining and greenspace, as well as redlining and birth outcomes, using sociodemographics at the time of HOLC maps as potential confounders. In this study, to examine the associations between historical redlining and present-day population burden of cardiovascular risk factors, we utilized historical census-tract-level sociodemographic data, present-day cardiovascular health data, and the HOLC maps data. We hypothesized that among neighborhoods subjected to HOLC grading in the 1930s, a less desirable grade would be associated with higher prevalence of three cardiovascular risk factor outcomes in 2020: obesity, hypertension, and diabetes.

## Results

Of the 6,981 census tracts with HOLC grades and outcome data, 330 were graded A (4.7%), 1,337 graded B (19.2%), 3,390 graded C (48.6%), and 1,924 graded D (27.6%; see Table 1). The sample covered 2,099 Midwest region tracts, 2,675 Northeast tracts, 704 South tracts, and 1,504 West tracts. There was good visual consistency between the 2020 tract-level grade boundaries and the original HOLC maps’ boundaries (Figure S1). The sensitivity analyses using an alternate centroid-based linking method and using 50% threshold produced grade boundaries that also largely agree with the original maps (Figure S1, Tables S1–S3). However, these analyses reduced the amount of census tracts included in the study and led to a smaller sample size.

**Table 1.** Data description of the 1940 sociodemographics, geographical distributions of HOLC grades, and cardiovascular risk factors.

Variable	*N*	HOLC grade A	HOLC grade B	HOLC grade C	HOLC grade D
1940 sociodemographics					
Black (%)	6,981	1.6 (2.4)	1.0 (2.5)	1.6 (6.4)	12.4 (23.8)
Non-White (%)	6,981	2.1 (3.5)	1.2 (2.8)	1.8 (6.4)	13.1 (23.9)
Foreign White (%)	6,981	9.5 (6.7)	13.3 (7.9)	16.2 (9.0)	19.2 (10.8)
Median home value (1k$)	6,981	8.8 (4.1)	6.1 (2.5)	4.3 (1.6)	3.3 (1.8)
College degree and above (%)	6,981	20.9 (7.4)	11.8 (7.0)	6.9 (5.4)	3.8 (3.5)
Unemployment (%)	6,981	5.3 (3.0)	8.9 (3.9)	13.0 (4.9)	20.0 (6.7)
Renter (%)	6,981	39.8 (20.0)	51.8 (20.6)	58.4 (19.0)	68.9 (17.8)
Population density (1 k/km^2^)	6,981	3.2 (6.8)	6.6 (8.5)	7.1 (7.4)	11.2 (11.4)
Risk factor					
Obesity	6,981	27.9 (7.8)	30.4 (8.7)	32.5 (9.1)	34.1 (9.5)
Diabetes	6,981	9.2 (3.7)	10.6 (4.2)	11.9 (4.5)	12.8 (5.3)
Hypertension	6,767	29.5 (7.1)	30.5 (8.1)	31.3 (8.9)	32.4 (10.3)
Region					
Midwest	2,099	124 (5.9%)	407 (19.4%)	1,042 (49.7%)	525 (25.0%)
Northeast	2,675	76 (2.8%)	528 (19.7%)	1,308 (48.9%)	763 (28.5%)
South	704	53 (7.5%)	144 (20.5%)	272 (38.6%)	235 (33.4%)
West	1,504	77 (5.1%)	258 (17.2%)	768 (51.1%)	401 (26.7%)
Total number by grade	6,981	330 (4.7%)	1,337 (19.2%)	3,390 (48.6%)	1,924 (27.6%)

The values in the four HOLC grade columns are mean values with standard deviations in parentheses for 1940 sociodemographics and risk factors, and the counts of tracts with percentage in parentheses for regions. Population density in 1940 was calculated using the population of the census tract in 1940 divided by the area of the census tract in 1940.

Across grades, the mean percentage of residents that were Black (or non-White) was generally low (below 15%), and grade D tracts exhibited substantially higher values compared with A, B, and C. For other sociodemographic variables, similar patterns emerged across grades. In tracts ranging from grade A to grade D, the mean values increased progressively in percent foreign White population, percent unemployment, percent renter, and population density, while both median home value and percent with a college degree and above declined. The 2020 prevalence for obesity, diabetes, and hypertension also increased from grade A to grade D. Grade D tracts had the highest mean prevalence of 34.1% for obesity, 12.8% for diabetes, and 32.4% for hypertension.

Table 2 shows the exploratory results of ordinary least squares (OLS) models that tested for prevalence differences in areas graded A by HOLC versus those that were graded B, C, or D; the four HOLC grades were included in one model. The three less desirable grade tracts (B, C, and D) were associated with a higher prevalence of diabetes, hypertension, and obesity compared with grade A tracts in unadjusted and region-adjusted models. However, this association was largely explained by the 1940 sociodemographics after adjustment. Table 3 presents OLS results comparing adjacent pairs of grades. Tracts graded as less desirable in the 1930s (B vs. A, C vs. B, D vs. C) tended to have higher prevalence of diabetes, hypertension, and obesity in 2020, but adjustment for sociodemographic differences in 1940 again absolved most of these differences.

**Table 2.** Estimates for prevalence differences for three cardiovascular risk factors across four HOLC grades (using grade A tracts as reference).

Outcome variables	I. Crude	II. Adjusted (regions)	III. Adjusted (socio-demographics and regions)
Grade B			
Diabetes	1.40 (0.84, 1.96)	1.61 (1.07, 2.15)	−0.16 (−0.72, 0.39)
Hypertension	0.95 (−0.16, 2.05)	1.54 (0.55, 2.54)	−0.26 (−1.29, 0.77)
Obesity	2.49 (1.40, 3.58)	3.60 (2.67, 4.54)	−0.52 (−1.47, 0.43)
Grade C			
Diabetes	2.71 (2.19, 3.24)	3.04 (2.54, 3.55)	0.41 (−0.18, 0.99)
Hypertension	1.76 (0.72, 2.79)	2.69 (1.76, 3.63)	0.18 (−0.91, 1.26)
Obesity	4.59 (3.57, 5.62)	5.94 (5.06, 6.82)	−0.02 (−1.03, 0.98)
Grade D			
Diabetes	3.63 (3.09, 4.18)	3.89 (3.37, 4.41)	0.35 (−0.31, 1.01)
Hypertension	2.93 (1.86, 4.00)	3.58 (2.62, 4.55)	0.08 (−1.15, 1.30)
Obesity	6.11 (5.05, 7.17)	7.42 (6.51, 8.33)	0.14 (−0.99, 1.27)

This table presents nine regression models with 95% CIs. Model 1 regresses diabetes prevalence on grade indicators B, C, and D, using grade A as the reference category. Model 2 adds region indicators as covariates. Model 3 further adjusts for 1940 sociodemographic variables in addition to region. Models 4–6 repeat this sequence of models for hypertension prevalence. Models 7–9 repeat the same sequence again for obesity prevalence. In the analysis, we used 6,981 census tracts for models on diabetes and obesity, and a subset of 6,767 tracts was used for models on hypertension.

**Table 3.** Estimates for prevalence differences for cardiovascular risk factors between tracts with lower HOLC grades and tracts with higher HOLC grades.

Outcome variables	*N* for whole sample (*N* for lower grade)	I. Crude	II. Adjusted (regions)	III. Adjusted (socio-demographics and regions)
Grade B vs. grade A				
Diabetes	1,667 (1,337)	1.40 (0.91, 1.89)	1.40 (0.92, 1.88)	−0.15 (−0.67, 0.37)
Hypertension	1,636 (1,312)	0.95 (−0.02, 1.91)	1.16 (0.24, 2.08)	−0.58 (−1.59, 0.43)
Obesity	1,667 (1,337)	2.49 (1.46, 3.52)	3.24 (2.35, 4.13)	0.16 (−0.78, 1.11)
Grade C vs. grade B				
Diabetes	4,727 (3,390)	1.32 (1.03, 1.6)	1.42 (1.15, 1.69)	0.59 (0.30, 0.89)
Hypertension	4,608 (3,296)	0.81 (0.26, 1.36)	1.12 (0.62, 1.63)	0.44 (−0.12, 1.00)
Obesity	4,727 (3,390)	2.10 (1.53, 2.67)	2.32 (1.83, 2.81)	0.37 (−0.16, 0.89)
Grade D vs. grade C				
Diabetes	5,314 (1,924)	0.92 (0.65, 1.19)	0.81 (0.56, 1.07)	−0.15 (−0.46, 0.15)
Hypertension	5,131 (1,835)	1.17 (0.63, 1.71)	0.81 (0.34, 1.29)	−0.20 (−0.76, 0.36)
Obesity	5,314 (1,924)	1.52 (1.00, 2.04)	1.45 (1.01, 1.89)	−0.17 (−0.68, 0.34)
Total *N*	6,981			

This table presents 27 regression models with 95% CIs. The first nine models focused on grades A and B tracts. Model 1 regressed diabetes prevalence on a lower grade indicator (1 = grade B, 0 = grade A) and a constant. Model 2 added region indicators. Model 3 further adjusted for the 1940 socio-demographics. Models 4–6 repeated this for hypertension, and models 7–9 repeated for obesity. The next nine models repeated this sequence, comparing grades B and C tracts. The final nine models repeated the comparisons between grade C and grade D tracts. This structured approach examined the association between classification as a lower grade and disease prevalence for each grade pair, with increasing adjustment for region and sociodemographic differences.

The results after inverse probability weighting (IPW; Table 4) demonstrated the prevalence differences (<1%) when comparing grades C and B tracts, while the prevalence of the three risk factors did not show the difference when comparing grades A to B or C to D tracts (*P* > 0.05). Specifically, grade C tracts had an expected 0.89% (95% CI: 0.48%, 1.29%), 0.83% (95% CI: 0.05%, 1.61%), and 0.90% (95% CI: 0.07%, 1.73%) higher prevalence of diabetes, hypertension, and obesity than grade B tracts. Additionally, results after full matching (Table S4) also show an expected higher prevalence (<1%) of diabetes, hypertension, and obesity in grade C tracts compared with grade B tracts, which further validates results from the IPW analysis. In contrast, no differences emerged for A and B or C and D comparisons after IPW or propensity score matching (*P* > 0.05). In summary, areas classified as grade C in the 1930s had modestly higher (<1%) expected disease prevalence in 2020 than those classified as grade B in the 1930s, while grades A and B and C and D comparisons showed no differences (*P* > 0.05).

**Table 4.** Effect estimates of prevalence difference of the three cardiovascular risk factors from IPW.

Risk factors	Trimmed *N* (original *N*)	Effect estimate (%)
Grade B vs. grade A		
Diabetes	1,583 (1,667)	0.27 (−0.56, 1.10)
Hypertension	1,552 (1,636)	0.22 (−1.40, 1.83)
Obesity	1,583 (1,667)	1.24 (−1.02, 3.50)
Grade C vs. grade B		
Diabetes	4,488 (4,727)	0.89 (0.48, 1.29)
Hypertension	4,373 (4,608)	0.83 (0.05, 1.61)
Obesity	4,488 (4,727)	0.90 (0.07, 1.73)
Grade D vs. grade C		
Diabetes	5,047 (5,314)	−0.24 (−0.83, 0.36)
Hypertension	4,867 (5,131)	0.03 (−1.13, 1.19)
Obesity	5,047 (5,314)	−0.17 (−1.25, 0.92)

This table presents results from nine MSMs after IPW, each with a 95% CI. The first three models focused on grade A and grade B tracts, the next three models focused on grade B and grade C tracts, and the last three models focused on grade C and grade D tracts, with the lower grade defined as the treatment. The models examined the prevalence difference for the three cardiovascular risk factors in 2020, estimating the percentage of cases in 2020 that would not have occurred if tracts were classified into a higher rather than a lower HOLC grade.

## Discussion

Our results demonstrate that tracts classified into a less desirable HOLC grade in the 1930s had, prior to controlling for sociodemographics, increased prevalence of cardiovascular risk factors in 2020, yet most of the results were not significant after adjustment for confounding by historical neighborhood demographics or when employing causal methods. The exception was a persistent effect in the prevalence of cardiovascular risk factors for “yellowlining” areas—suggesting a potential causal impact for the B vs. C grades. In other words, those which were classified as grade C had a higher prevalence of diabetes, hypertension, and obesity in 2020 than those graded B. As many previous studies have used present-day sociodemographic variables as confounders (17), our study improves upon previous analysis by using more relevant antecedents of HOLC grading as potential confounders (i.e. sociodemographic factors in 1940), using IPW to create a pseudo-randomized experiment to increase exchangeability.

Areas graded C by HOLC exhibited a higher prevalence of cardiovascular health risk factors in 2020 compared with those graded B. In comparison, we did not find a significant difference for A and B and C and D grades. This suggests that “yellowlining” (as grade C was in color yellow), instead of “redlining,” might have played a more substantial role than previously thought. This is consistent with two other studies—Aaronson et al. (1) found that a less desirable grade for B versus C was associated with reduced homeownership rates; the direction of these associations reversed for C versus D after 1970/1980, but not for B versus C. Similarly, Nardone et al. (25) also found elevated odds of preterm birth and size for gestational age for grade C neighborhoods compared with grade B neighborhoods, while they found reduced odds of preterm birth, small-for-gestational age, and low birth weight for grade D neighborhoods versus grade C neighborhoods.

There are several possible explanations (1, 25): first, compared with C areas, D areas may have experienced more redevelopment and gentrification due to factors like proximity to the urban center and older housing structure (7). In the late 20th century, urban renewal disproportionately displaced Black neighborhoods and previously redlined neighborhoods (15); Second, D areas have higher proportion of minority communities, and policies in the following decades may have successfully addressed inequity in D areas rather than C areas—specifically, since the 1960s, the Fair Housing Act and other related statutes, regulations, and executive orders (27) may have helped address the inequities for neighborhoods mostly concentrated in grade D areas; third, D areas may have already suffered more restrictive lending and other discriminatory housing practices pre-HOLC mapping, thus the marginal impact was more pronounced in C areas after the HOLC maps were made.

To conclude, when investigating obesity, hypertension, and diabetes, B and C difference, rather than C and D or A and B difference, may play a more pronounced role in enhancing other chronic stressors and neighborhood stigma resulting from various factors such as discrimination, poverty, and lack of other economic, social, and healthcare resources. Drawing from the structural sorting perspective, the segregation in these neighborhoods likely continued due to subsequent policies and institutionalized efforts, reinforcing a cycle of self-perpetuating segregation (15). Thus, policy interventions may need to address the disparities from overlooked neighborhoods resulting from historical “yellowlining” practices.

Our study relies on HOLC maps to measure redlining practices, but these maps may not have accurately captured the actual redlining practices, thus introducing the potential for exposure measurement error. The degree to which the FHA, other government programs (including HOLC itself), and private mortgage lenders relied on the HOLC maps in decision-making has been widely disputed due to insufficient historical evidence (28–30). This analysis was conducted under two assumptions about the maps’ influence/meaning: (i) HOLC maps institutionalized racism and influenced discriminatory lending and foreclosure practices conducted by the FHA, HOLC, and private lenders; or (ii) instead of direct impacts, HOLC maps simply served as a proxy for discriminatory practices in the housing market of the time (2, 6, 7). Supporting the first view, scholars have argued that HOLC maps were widely referenced by private lenders and government institutions such as FHA with “ample evidence” (31, 32). In addition to lending practices, HOLC maps may have informed foreclosure ownership decisions (29, 33) as well as higher interest rates (30). This argument is also supported by data-driven findings-based on boundary design and propensity score methods, neighborhoods receiving less desirable grades in the HOLC maps subsequently resulted in lower rates of home ownership, reduced property values and rents, along with racial segregation in the following decades (1); cities evaluated by HOLC experienced higher segregation compared to those left unassessed (15).

On the other hand, scholars who argue against the impacts of HOLC maps say that firstly, the majority of the HOLC loans were already distributed before HOLC maps were made (33), and secondly, HOLC provided many loans in grade C and grade D areas when compared with FHA, thus the FHA was more obviously engaged in discriminatory lending (29). And thirdly, HOLC maps were not shared widely with other government institutions and private lenders (30). FHA produced its own “redlining” maps to guide their lending practices; however, most evidence was likely destroyed due to lawsuits (33). Banks were also asked to draw their own maps that might have essentially replicated the HOLC maps (1, 31). A recent study demonstrated that, for tracts in Chicago rated worst by the FHA, the effects of discriminatory lending were more pronounced in terms of home values and ownership rates when compared with the HOLC map, suggesting that HOLC maps might not fully capture the extent of areas most affected (7). In other words, relying solely on HOLC maps may not reveal the full extent of disparities resulting from discriminatory lending practices, especially for discriminatory lending based on FHA maps. This potential measurement error due to any misclassification of the exposure is likely to be nondifferential, as it could affect all categories. However, even random nondifferential misclassification can cause bias away from the null, potentially leading to a biased estimate of the association between HOLC grades and health outcomes. Even so, our analysis remains valuable, as Mujahid et al. (6) suggested that HOLC maps are a useful, albeit imperfect, data source for studying historical housing discrimination, given the constraints of data availability from that era. These maps likely captured some of the discriminatory mortgage lending practices of the 1930s and 1940s, even if they did not fully represent the extent at the time. Additionally, despite its relatively modest scale and resources, the HOLC intervention fostered “self-reinforcing” dynamics in racially biased policymaking and “self-perpetuating” segregation in urban mobility, leading to enduring effects (15).

It is important to note that the study used comparisons between HOLC grades for prevalence differences rather than analyzing the actual prevalence values, which were still quite high in C and D areas in 2020 and require intervention. The finding that grade D tracts did not markedly differ from grade C tracts reflects in part a lack of statistical power to detect differences between those groups. Additionally, the research methods used in this study are not able to determine if there were differences in prevalence between grade B tracts and grade D tracts. As the use of HOLC maps in practice was disputed, our estimates might not fully capture the true impacts of discriminatory lending practices due to exposure measurement error, especially if the boundaries of HOLC maps were different from those used by FHA and private lenders. The method used for linking HOLC grades to tracts cannot account for tracts that overlap with multiple grades, which may impact the results, and comparisons with other research on this subject should consider variations in how HOLC grades are linked to census tracts. Additionally, our study's scope was constrained to areas with accessible data. This may have resulted in the exclusion of certain areas that experienced redlining, or areas lacking complete 1940 socioeconomic data. For example, tracts that lacked complete 1940 socioeconomic data had a higher proportion of grade A tracts compared with those with complete 1940 socioeconomic data. This discrepancy could potentially introduce a form of selection bias in our analysis. Notably, the outcomes from CDC PLACES were modeled prevalence estimates, based on small-area estimation techniques that relied heavily on the demographic characteristics of the tract (34). Since the discriminatory lending practices likely have an impact on demographics (15), the presented associations may result from racial/ethnic composition in the census tracts. While our study intentionally did not adjust for current sociodemographic factors, as they are not considered confounders in our analysis, we acknowledge there could be potential bias if the modeling process used by PLACES does not fully account for the true variability in health outcomes associated with these contemporary sociodemographic characteristics. Additionally, in the 2020 health data, the actual onset of diabetes and hypertension among residents across their lifetimes remains unknown. Furthermore, the census tract-level analysis risked ecological fallacy and obscured individual-level risk factors. Other unmeasured confounders, due to data availability constraints, were not included in this study. To build on this research, future studies could examine the full timeline from the 1930s onwards and employ individual-level directly measured outcome data.

Our findings revealed an association between less desirable HOLC grades from the HOLC maps in the 1930s and increased prevalence of cardiovascular risk factors in 2020. While most results were statistically nonsignificant after IPW, the “yellowlining” B and C grade difference emerged as a notable risk differentiator in the present-day prevalence of cardiovascular health disparities. Further place-based policy interventions are still needed to mitigate and ultimately eliminate the potential present-day impacts of these historical policies.

## Materials and methods

### Outcomes

We examined the current prevalence of three cardiovascular disease risk factors at the census tract level in areas graded by HOLC in the 1930s: obesity and diabetes in 2020, and hypertension in 2019. Outcome data were created by the Centers for Disease Control and Prevention (CDC) PLACES project, 2022 release. For outcome data from PLACES, only crude measures (without adjustment for demographic age distributions) were available at the census tract level. Note that the outcome data were modeled estimates, which used small-area estimation techniques that were dependent on the demographic characteristics of the tract (25). Though PLACES provided the outcome data for most US census tracts, not all tracts have both HOLC grades and cardiovascular risk prevalence (i.e. the outcome data) information. Therefore, the sample sizes of the three risk factors vary slightly due to data availability. In total, we selected 6,981 census tracts for prevalence of obesity and of diabetes, spanning 63 counties in 27 states. For hypertension prevalence, we included a subset of those tracts, totaling 6,767, due to missing hypertension prevalence data in some areas (e.g. New Jersey).

### Exposure

We used HOLC map data from the Mapping Inequality project (10). We selected four grades from the HOLC maps: A, B, C, and D, representing “best,” “still desirable,” “declining,” and “hazardous,” respectively. Due to heterogeneity in the classification's meaning across places, we excluded E graded areas from our analyses. We also did not include areas that were left ungraded by the HOLC in this study.

We first linked HOLC grades (initially including grade E) to 2020 census tracts based on geographic overlap. We overlaid the 2020 census tract boundaries with boundaries of the five HOLC grades. Among 84,414 census tracts across the United States, 16,599 overlapped with HOLC graded areas. We then removed ∼22% of the 16,599 census tracts that had an overlay area of <25%, leaving us with 12,853 census tracts with HOLC grade information. We assigned to each tract the HOLC grade that covered the most land area in that tract, a method similar to previous studies (Equation 1) (6). We conducted a sensitivity analysis using 50% as the threshold. We also employed a sensitivity analysis to link the HOLC grades to census tracts based on where the centroid of the tracts located in the HOLC map (35).


(1) $$\mathrm{Grade}(t)=\arg\;\underset{g\in G}{\max}\;\left\{\sum_{s\;\mathrm{in}\;t,\mathrm{Grade}(s)\;=\;g}\mathrm{area}(s)\right\},$$

where Grade(*t*) is the assigned grade for census tract *t*; *G* is the set of all possible grades. *G* = {*A*, *B*, *C*, *D*}; *g* is a particular grade in the set *G*; *s* is the subtract in tract *t*. A tract *t* may have several subtracts that were divided by the boundaries of HOLC grades; area(*s*) is a function that returns the percentage of area the subtract *s* has in the tract *t*; and argmax is an operation that returns the argument of the maximum value of a function.

Then, we linked 2020 census tracts with 1940 sociodemographic data using 7,563 census tracts from the 1940 National Historical Geographic Information System (NHGIS) dataset (36). Considering that 1940 census tracts were generally larger in size than those in 2020, our criterion for linking was whether the centroid of a 2020 tract fell within a 1940 tract. Using this method, we further narrowed our sample from the initial 12,853 census tracts to 7,971 tracts. These 7,971 tracts had both 1940 sociodemographic data and HOLC grade data. We removed tracts with grade E and obtained 7,967 tracts. After further integrating this dataset with the CDC PLACES data, we removed tracts with missing health outcome information and obtained the final sample sizes for each health outcome as described in the Outcomes section. For a detailed understanding of the process, please refer to the flow chart in Figure S2.

### Potential confounders

We selected sociodemographic measures from 1940 in the HOLC mapping areas to serve as potential confounders—for instance, the percentage of the population in the neighborhood that was Black could influence the assignment of HOLC grades; it could also be a marker for systemic racism in forms of greater poverty, poorer educational systems, diminished access to healthcare, worse living environment, and so on that may be linked to the later prevalence of the three cardiovascular disease risk factors. Based on the availability of the historical sociodemographic data, we selected the following variables as potential confounders from the Integrated Public Use Microdata Series NHGIS (36): percent Black population, percent non-White population, percent foreign White population, median home values, percent adults 25 years and older with college degree and above out of the total population, percent unemployed among adults 25 years and older out of the total population, percent tenant-occupied dwelling units of total dwelling units, and population density. We used 1940 sociodemographic data instead of data from the 1930s for three reasons: the HOLC maps were produced in the late 1930s; neighborhood sociodemographic profiles were changing substantially during the Great Depression beginning in 1929, such as rising unemployment rates, migration, foreclosures (37), thus the 1940 data might better represent the circumstances during which the HOLC maps were drafted; and 1940 data provide greater geographic coverage than 1930 data (26). Nevertheless, the intense migration and changing sociodemographics, especially from 1935 to 1940 introduce uncertainty in the construction of IPW weights. Thus, it is important to interpret our findings with caution. The HOLC maps might be more appropriately viewed as proxies for the discriminatory housing market practices of that era, rather than as direct determinants of lending practices. Additionally, region—Midwest, Northeast, South, and West—was also included as a potential confounder, with the assumption that different socio-, cultural, and political contexts in different regions may have impacted discriminatory housing practices at the time, as well as current day cardiovascular health outcomes.

### Analyses

We conducted three statistical analysis steps to examine the association between HOLC grades and the selected cardiovascular risk factors. First, as an exploratory analysis, we used OLS regression to evaluate the association between outcome prevalence and the four HOLC grades, using grade A as the reference category and with B, C, and D included in the same model. Second, to compare differences between grades, we used OLS models to investigate how the prevalence of obesity, hypertension, and diabetes varies across each pair of the grades (A and B, B and C, C and D). Thus, each model only includes two grades. The higher grade in each pair was used as the reference category, coded as 0. For example, when comparing grades A and B, grade A was the reference category coded as 0 and grade B was the less desirable grade coded as 1. For each of the two steps, we ran unadjusted models, models adjusting for region, and models adjusting for both region and 1940 sociodemographics. This allowed us to thoroughly investigate the relationships between HOLC grades and the prevalence of the three selected risk factors.

Third, to evaluate the prevalence difference between less desirable HOLC grades and more desirable HOLC grades for diabetes, hypertension, and obesity, we used IPW and marginal structural models (MSM) for each pair of the grades (A and B, B and C, C and D). We first calculated propensity scores for each census tract using the 1940 sociodemographics and region to predict the likelihood a tract was classified into the less desirable grade in each pair (A and B, B and C, C and D) using a logit model. We trimmed the sample by 2.5% on both ends of the propensity score distribution in order to avoid extreme values. We then used IPW to create a pseudo-population. Finally, we employed MSMs on the pseudo-population to estimate the difference in the prevalence of diabetes, hypertension, and obesity had tracts been classified as a less desirable rather than a more desirable HOLC grade. As a sensitivity analysis, we implemented propensity score matching based on the previously calculated propensity scores. We used the MatchIt package in R for full matching to estimate the prevalence difference.

## Supplementary Material

pgae301_Supplementary_Data

## Data Availability

All data and codes are publicly available at https://doi.org/10.5281/zenodo.11660839.
