# Supplementary material for: Associations between 1930s HOLC grades and estimated population burden of cardiovascular disease risk factors in 2020
Source: PNAS Nexus. 2024 Aug 9;3(8):pgae301. doi: 10.1093/pnasnexus/pgae301 (PMC11323776; doi:10.1093/pnasnexus/pgae301)
Supplement: pgae301_Supplementary_Data [file pgae301_supplementary_data.zip › PNASNEXUS-PNASNEXUS-2024-00242-TR-s01.docx]

**Supporting Information for**

Associations between 1930s HOLC Grades and Estimated Population Burden of Cardiovascular Disease Risk Factors in 2020

Hanxue Wei^a^, Benjamin R. Spoer^a^, Andrea R. Titus^a^, Taylor M. Lampe^a^, Marc N. Gourevitch^a^, Jacob W. Faber^b,c^, Steven J. Korzeniewski^d^, Samantha J. Bauer^d^, Lorna E. Thorpe^a^

^a^Department of Population Health, New York University Grossman School of Medicine, New York, NY, USA.

^b^Robert F. Wagner School, New York University, New York, NY, USA.

^c^Department of Sociology, New York University, New York, NY, USA.

^d^Department of Family Medicine and Public Health Sciences, Wayne State University School of Medicine, Detroit, MI, USA.

Lorna E. Thorpe

**Email:**  [Lorna.Thorpe@nyulangone.org](mailto:Lorna.Thorpe@nyulangone.org)

**This PDF file includes:**

Supporting text

Figures S1 to S3

Tables S1 to S4

SI References

Supporting Information Text

**Methods**

**Limitations of HOLC map data.** We found 189 overlaps in polygons in the original HOLC maps shapefile from the Mapping Inequality project (Robert et. al., 2023) that were probably due to inaccuracies in data input process. The overlaps were generally small in size with little impact on our main analysis. In our main analysis of assigning HOLC grades to tracts, we ignored the overlaps as our analysis used areas. In our sensitivity analysis, the overlaps affected four 2020 census tracts when joining tracts with HOLC maps. We dropped the four census tracts. (For instance, the centroid of tract with GEOID ‘47157003500’ was within HOLC grade A and B at the same time in Memphis.)

**HOLC grade assignment.** In assigning HOLC grades to 2020 census tracts, we identified tracts where the difference between the proportions of largest and second-largest area grades was less than 5%. This scenario occurred in 368 out of the 6,981 tracts observed in this study, accounting for approximately 5% of the total tracts. We uniformly classified these tracts using our established method, assigning to each tract the HOLC grade that covered the largest land area within each tract (as per Equation 1). As such, we acknowledge a methodological limitation in our approach to assigning HOLC grades due to potential inaccuracies in grade assignment when there is a tie, affecting a small portion of the total tracts. Furthermore, our method of assigning the grade with the largest proportion in each tract as the tract's HOLC grade improves upon previous analyses that utilized geographically-weighted averaging systems (Linde et al., 2023). For example, such an approach might assign a middle grade, such as B, to a tract that predominantly consists of the highest (A) and lowest (D) grades, potentially misrepresenting the tract's characteristics. In contrast, our method ensures that tracts are only assigned grades that were assigned to areas located within tract boundaries, improving the accuracy of our grade allocation.

The chosen threshold of keeping census tracts with an overlay area above 25% in relation to HOLC grades represents a compromise between preserving sufficient data and ensuring accuracy. In our sensitivity analysis, we included a 50% threshold as well, which resulted in an 18% reduction in the sample size for comparison. Our main model analyses used the sample generated from the 25% threshold.

**Geographical unit.** We opted not to use state-level units in our propensity score models to avoid overfitting.

| 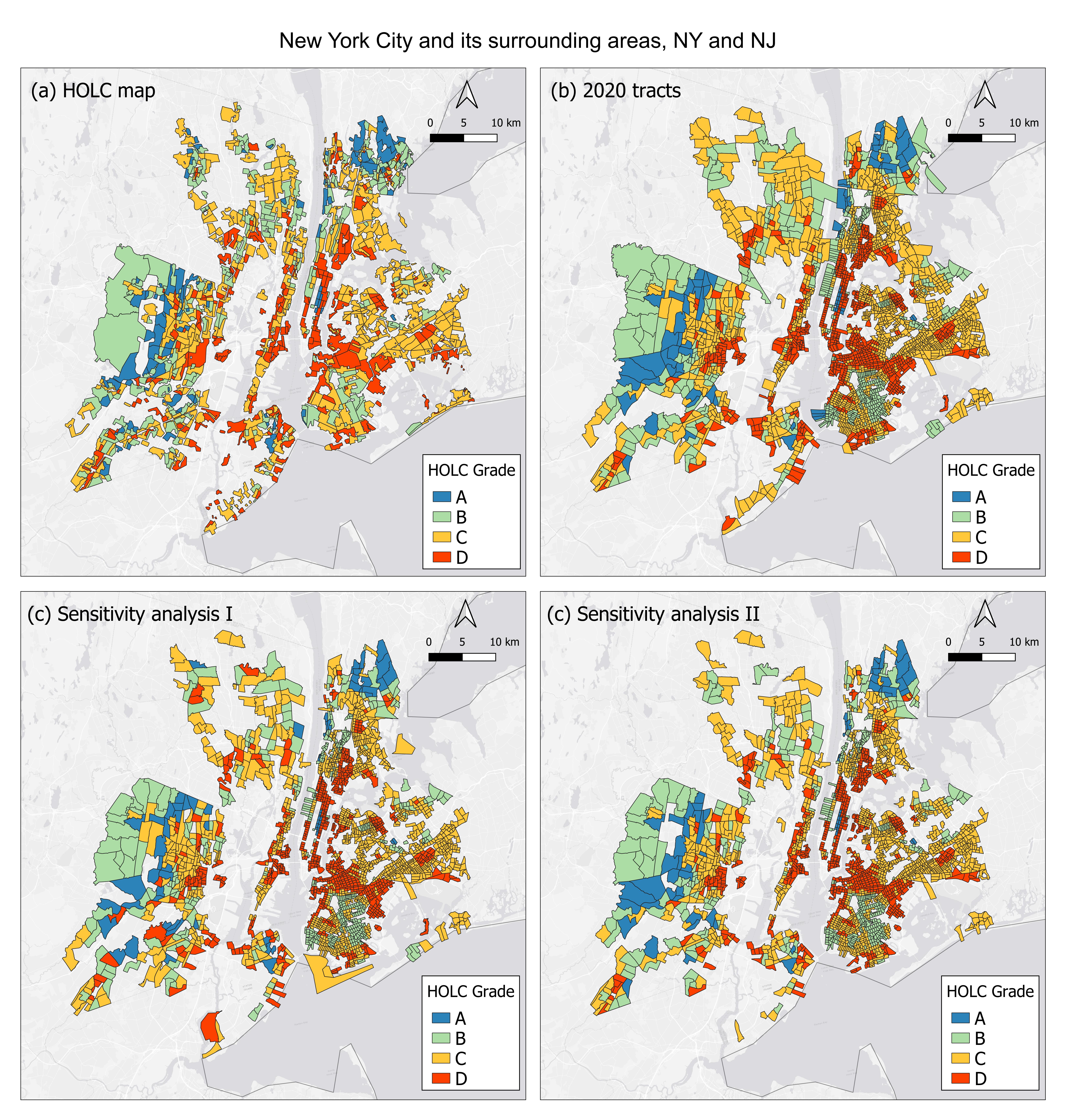  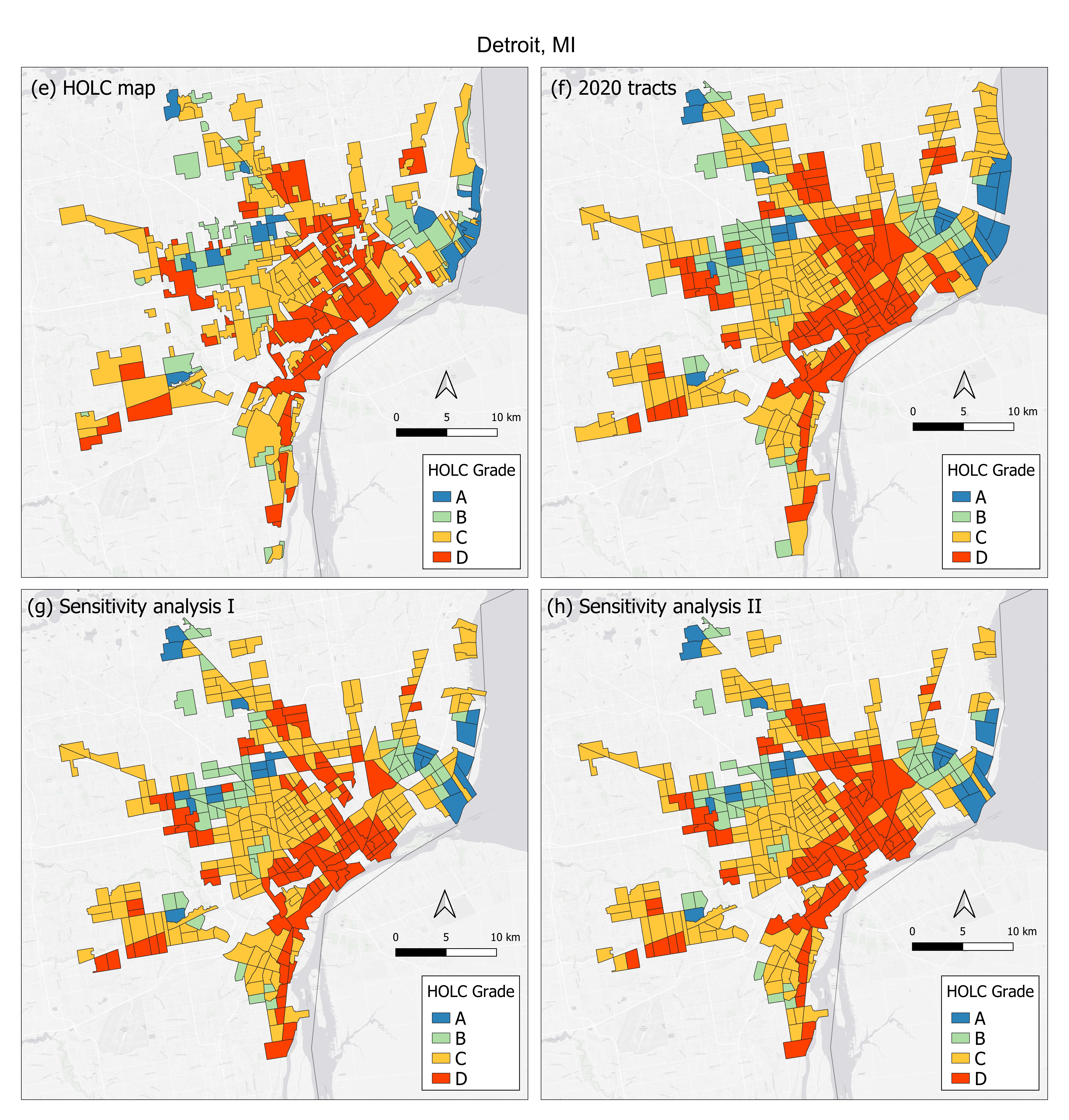 |
| --- |

Fig. S1. Assignment of HOLC grade to census tracts using in and around New York City area and Detroit area as examples. Figure (a) and (e) show the original HOLC maps (excluding grade E); Figure (b) and (f) show the 2020 census tracts with assigned 1940 HOLC grades in the main analysis (using 25% threshold); Figure (c) and (g) show the sensitivity analysis I using centroid to link the 2020 tracts with 1940 grades; Figure (d) and (h) show the sensitivity analysis II using 50% threshold to link the 2020 tracts with 1940 grades.


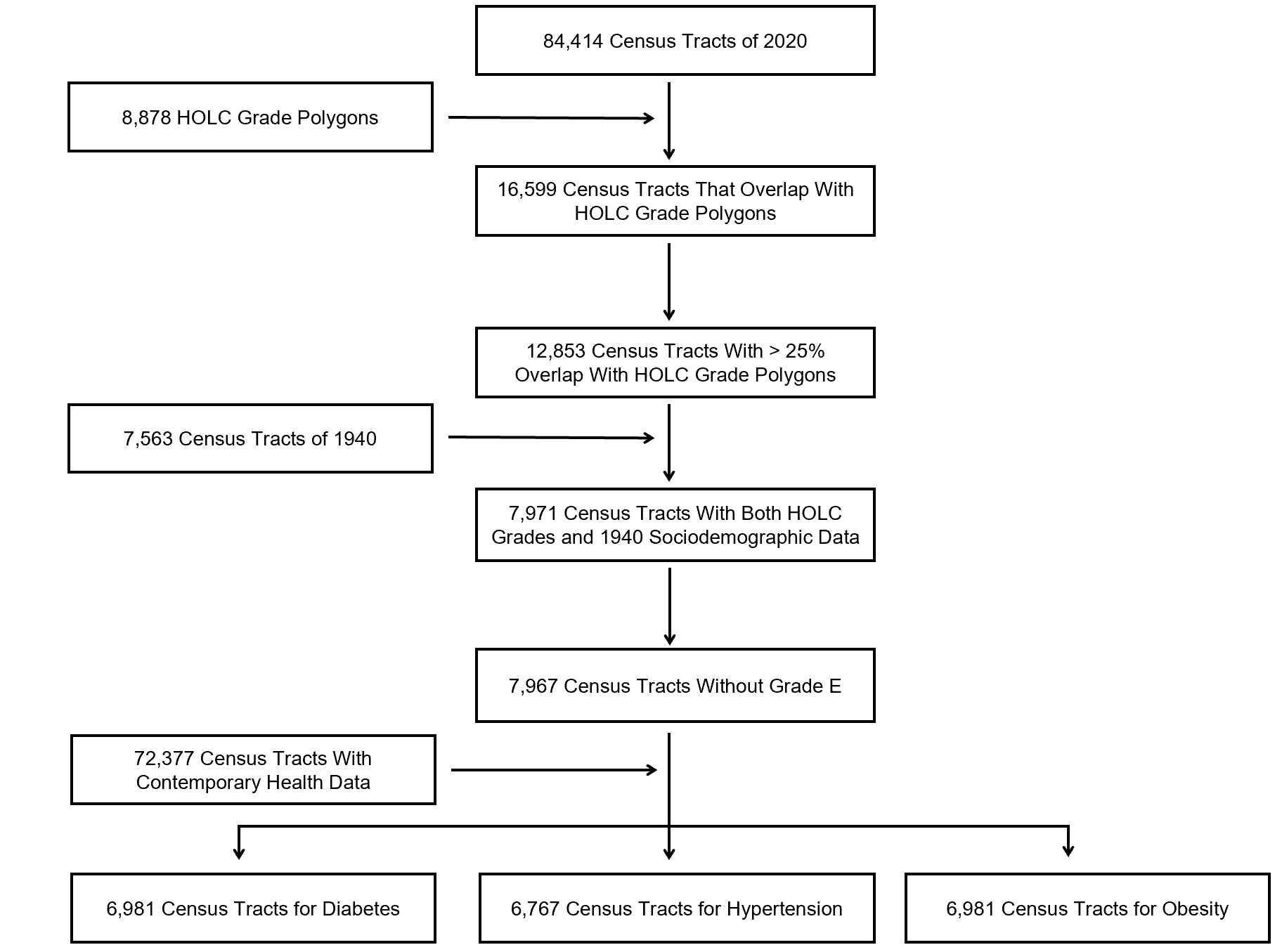


**Fig. S2.** Inclusion protocol for data used in models.

| A v B  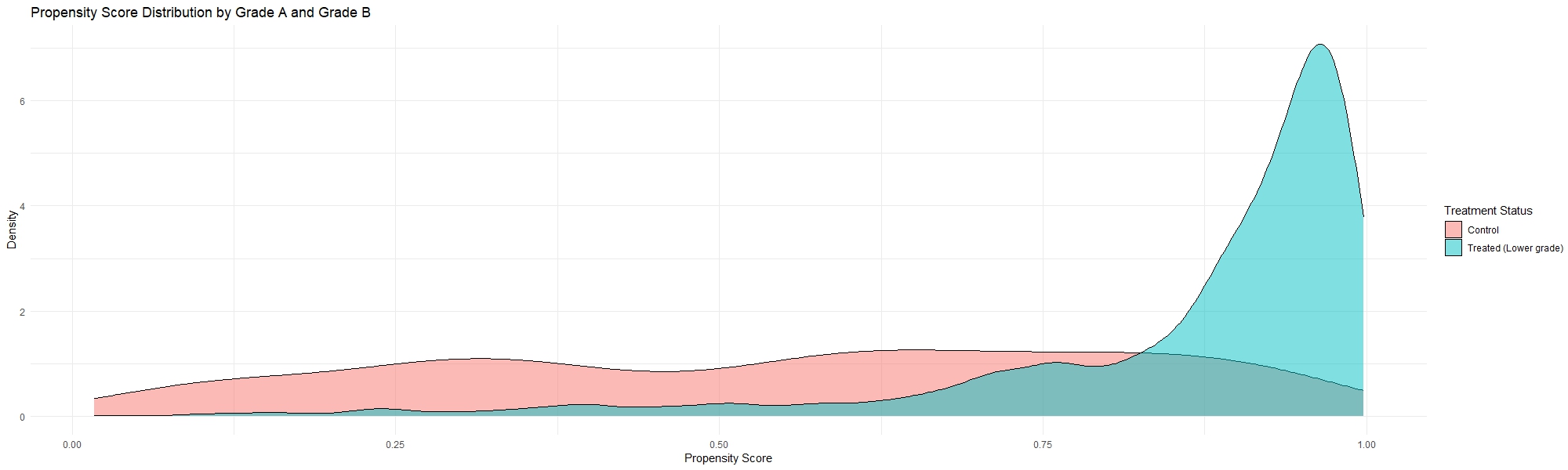 |
| --- |
| B v C  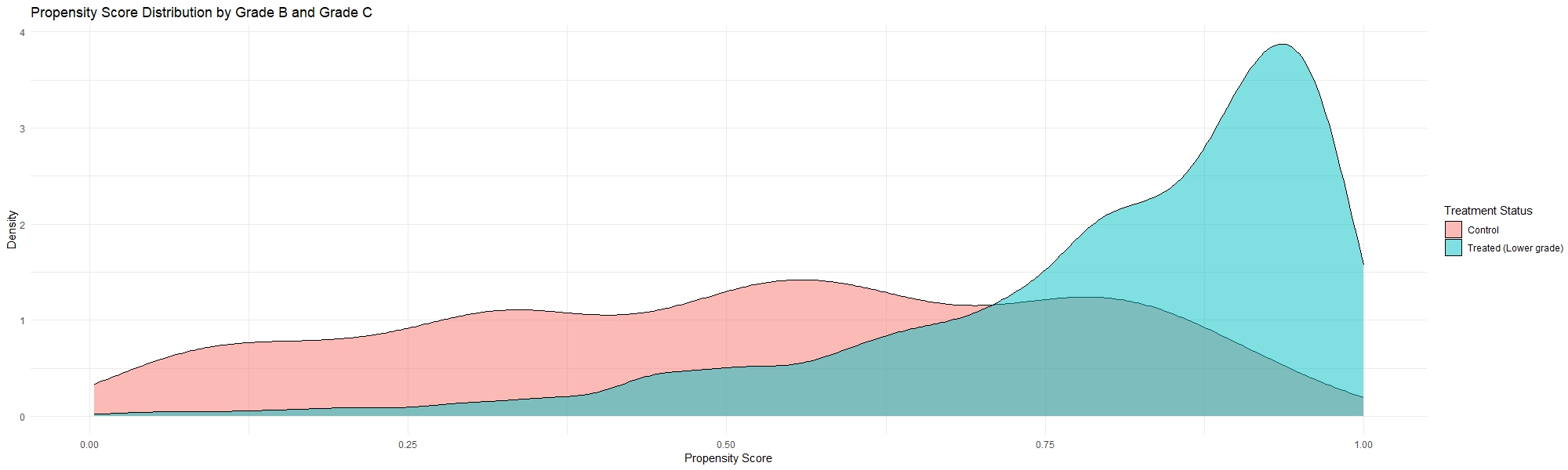 |
| C v D  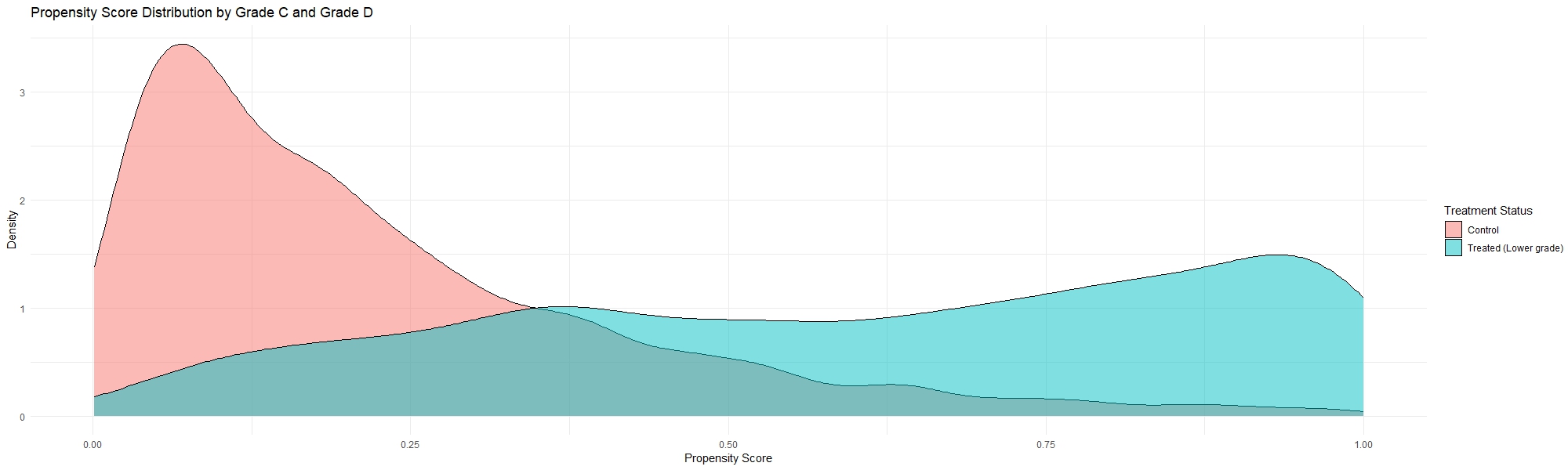 |

Fig. S3. Propensity score distribution for A & B, B & C, or C & D. Blue color represents the lower grade.

Table S1. The distribution of 2020 census tracts by HOLC grade.

| Grade | Number of census tracts | Percentage |
| --- | --- | --- |
| A | 795 | 6.2% |
| B | 2,568 | 20.0% |
| C | 5,873 | 45.7% |
| D | 3,609 | 28.1% |
| E | 8 | 0.1% |
| Total | 12,853 | 100.00% |

Table S2. Sensitivity analysis: the distribution of 2020 census tracts by HOLC grade using the centroid method.

| Grade | Number of census tracts | Percentage |
| --- | --- | --- |
| A | 686 | 6.4% |
| B | 2,209 | 20.5% |
| C | 4,858 | 45.0% |
| D | 3,037 | 28.1% |
| E | 7 | 0.1% |
| Total | 10,797 | 100% |

The centroid method was generally considered less accurate than other area-based methods, because the irregular shapes of tracts and HOLC grades, as well as cases when a tract overlapped with multiple HOLC grades, made this method less reliable (Noelke et al., 2022).

Table S3. Sensitivity analysis: the distribution of 2020 census tracts by HOLC grade using 50% threshold.

| Grade | Number of census tracts | Percentage |
| --- | --- | --- |
| A | 623 | 5.9% |
| B | 2,118 | 20.1% |
| C | 4,890 | 46.3% |
| D | 2,921 | 27.7% |
| E | 7 | 0.1% |
| Total | 10,559 | 100% |

Table S4. Effect estimates of prevalence difference of the three cardiovascular risk factors from propensity score matching.

| Risk factors | Matched N | Effect Estimate (%) |
| --- | --- | --- |
| Grade B vs. Grade A | | |
| Diabetes | 1,667 | -0.26 (-1.38, 0.85) |
| Hypertension | 1,636 | -1.23 (-3.50, 1.04) |
| Obesity | 1,667 | 0.77 (-1.52, 3.06) |
| Grade C vs. Grade B | | |
| Diabetes | 4,727 | 0.93 (0.46, 1.40) |
| Hypertension | 4,608 | 0.92 (0.00, 1.84) |
| Obesity | 4,727 | 0.99 (0.05, 1.92) |
| Grade D vs. Grade C | | |
| Diabetes | 5,314 | 0.24 (-0.39, 0.88) |
| Hypertension | 5,131 | 0.88 (-0.43, 2.18) |
| Obesity | 5,314 | 0.57 (-0.59, 1.74) |

This table presents results from propensity score matching, each with a 95% confidence interval. For matching, we used full matching from the MatchIt package in R to obtain a more balanced dataset. The confidence intervals were calculated based on cluster-robust standard error (Abadie & Spiess, 2022). The first three models focused on grade A and B tracts, the next three models focused on grade B and grade C tracts, and the last three models focused on grade C and grade D tracts, with the lower grade defined as the treatment. The models examined the prevalence difference for the three cardiovascular risk factors in 2020, estimating the percentage of cases in 2020 that would not have occurred if tracts were classified into a higher rather than a lower HOLC grade.

**SI References**

1. Noelke, C., Outrich, M., Baek, M., Reece, J., Osypuk, T. L., McArdle, N., ... & Acevedo-Garcia, D. (2022). Connecting past to present: examining different approaches to linking historical redlining to present day health inequities. PloS one, 17(5), e0267606.
2. Abadie, A., & Spiess, J. (2022). Robust post-matching inference. Journal of the American Statistical Association, 117(538), 983-995.
3. Linde S, Walker RJ, Campbell JA, Egede LE. Historic Residential Redlining and Present-Day Social Determinants of Health, Home Evictions, and Food Insecurity within US Neighborhoods. J Gen Intern Med. 2023 Nov;38(15):3321-3328. doi: 10.1007/s11606-023-08258-5. Epub 2023 Jun 9. PMID: 37296361; PMCID: PMC10255945.
